# Aberrant inter-network functional connectivity in drug-naive Parkinson’s disease patients with tremor dominant and postural instability and gait difficulty

**DOI:** 10.3389/fnhum.2023.1100431

**Published:** 2023-02-02

**Authors:** Qi Wang, Miao Yu, Lei Yan, Jianxia Xu, Yajie Wang, Gaiyan Zhou, Weiguo Liu

**Affiliations:** ^1^Department of Neurology, Affiliated Brain Hospital of Nanjing Medical University, Nanjing, China; ^2^Department of Neurology, Affiliated Hospital of Jiangsu University, Zhenjiang, China

**Keywords:** Parkinson’s disease, tremor dominant, postural instability and gait difficulty, independent component analysis, resting state

## Abstract

**Background:** Insight into neural mechanisms of tremor dominant (TD) and postural instability and gait disorder (PIGD) subtypes in Parkinson’s disease (PD) is vital for understanding pathophysiological hypotheses underlying this phenotype. However, network disturbances and their correlation with motor subtypes of PD remain unclear. We aimed to investigate the alterations of intra- and inter-network functional connectivity (FC) in drug-naive PD patients with different motor subtypes.

**Methods:** Resting-state functional magnetic resonance imaging was performed on 25 drug-naive PD patients with TD (PD-TD) and 40 drug-naive PD patients with PIGD (PD-PIGD), and 37 healthy controls (HCs) underwent. The following networks were extracted using independent component analysis: sensorimotor network (SMN), left executive control network (LECN), right executive control network, anterior salience network (aSN), posterior salience network (pSN), ventral attention network (VAN), dorsal attention network (DAN), default mode network (DMN), visual network, and auditory network (AN). We measured FC values within and between these networks.

**Results:** There were no detectable variations in intra-network FC. PD-PIGD group demonstrated lower FC between aSN and pSN, as well as between VAN and DMN, in contrast to PD-TD group. Particularly, the FC strength between VAN and DMN was positively correlated with TD and tremor scores, and the best fitting classification models of TD and PIGD subtypes were based on the FC between aSN and pSN. Compared with HCs, both PD-TD and PD-PIGD patients displayed decreased FC between two SMN subnetworks, while PD-TD patients exhibited increased FC between the SMN subnetwork and pSN, and between LECN and VAN. Furthermore, PD-PIGD patients demonstrated decreased FC between the SMN subnetwork and AN.

**Conclusions:** The altered FC between aSN and pSN can be an imaging marker to distinguish PD-TD from PD-PIGD. We for the first time disclosed that the PD-TD patients compensated by increasing attention resources and the PD-PIGD patients displayed reduced FC between SMN and AN. Our findings provide a basis for identification and precision treatment of PD motor subtypes.

## Introduction

Parkinson’s disease (PD) is a highly heterogeneous neurodegenerative disorder characterized by a variety of motor (e.g., resting tremors, rigidity, postural disabilities) and non-motor symptoms (e.g., sleep disorders, pain, cognitive impairment, dysautonomia; Weintraub et al., 2020). Research has indicated that PD can be divided into different subtypes according to clinical manifestations, pathological mechanisms, progression patterns, and imaging markers (Berg et al., 2021). Classic and widely used classification of motor symptoms includes tremor dominant (TD), indeterminate, and postural instability and gait difficulty (PIGD; Jankovic et al., 1990). Patients with the PIGD subtype have a faster rate of disease progression than those with the TD subtype, are less sensitive to levodopa, are more likely to experience dyskinesia and motor fluctuations, and have a heavier burden of non-motor symptoms (Marras and Chaudhuri, 2016; Beretta et al., 2019). Importantly, further investigation on subtype-specific diagnosis and monitoring indicators can reveal the underlying mechanisms of neurodegeneration and guide targeted therapy. Large-scale brain networks and their connections may be critical to neural functional activities, according to previous functional magnetic resonance imaging (fMRI) research, and the disruption of these networks’ interaction patterns is linked to the occurrence of numerous neuropsychiatric disorders (Chand et al., 2017). Unfortunately, the neural mechanisms of PD heterogeneity are unclear, few studies have analyzed the functional degradation of TD and PIGD at the large-scale brain network level.

PD and other neuropsychiatric diseases are currently being diagnosed and monitored using resting-state fMRI (RS-fMRI), a non-invasive neuroimaging approach without completing any task to reflect global and local aspects of brain function (Cerasa et al., 2016; Zhang et al., 2017; Caspers et al., 2021). Previous studies have shown that different PD motor subtypes exhibit variations in resting-state functional connectivity (FC). In PD patients, PIGD symptoms have been demonstrated to be associated with gray matter atrophy and decreased FC in motor-related regions (Rosenberg-Katz et al., 2013). Motor and cognitive impairments showed a slower decline in PD patients with TD, which may be related to FC between the putamen and cerebellum (Shen et al., 2020).

Previous studies have demonstrated the neural basis of different motor subtypes of PD from the perspective of functional separation. Assessing FC using defined brain regions as seeds have significant limitations, with the results depending on the seed selection. Independent component (IC) analysis (ICA) is an influential data-driven brain imaging analysis technique without prior seed regions, offers an effective approach that provides an effective means for identifying functional systems within the brain during rest, which are commonly referred to as “resting-state networks” (RSNs) or “intrinsic connectivity networks” (ICNs; Beckmann et al., 2005). The spatial representation of the brain networks is represented by the ICA components extracted by enforcing orthogonality, while the FC correlation values between each network pair reflect their interaction. Emerging evidence revealed that reduced FC within default mode network (DMN) areas and between DMN and salience network (SN) is associated with somatic symptom disorder in PD patients (Franciotti et al., 2019). Compared to HCs, both PD patients with and without freezing of gait (FOG) displayed reduced network connections mainly involving the sensorimotor cortex, DMN, auditory network (AN), visual network (VN), subcortical regions, dorsal attention network (DAN), and limbic network (Ruan et al., 2020). A comprehensive investigation indicated that the triple network may contribute to the emergence of depression in PD (Liao et al., 2021). We noticed that these studies used the ICA method to detect brain network dysfunction in PD patients in relation to their clinical defects. Therefore, it is imperative to identify FC within and between large-scale brain networks in order to better understand the neural mechanisms of motor subtypes of PD.

In this study, we aimed to identify the relationship between abnormal brain networks and clinical variables in PD patients with TD and PIGD subtypes by using RS-fMRI and ICA to thoroughly evaluate FC within and between RSNs. Considering that prior research demonstrated the remodeling influence of antiparkinsonian medications, such as levodopa on the brain networks (Ko et al., 2015; Chen et al., 2021), we concentrated on drug-naive PD patients with TD and PIGD subtypes to eliminate the influence of medication. We hypothesized that: (1) PD patients with TD and PIGD subtypes would show different intra- and inter-network FC; and that (2) aberrant brain network FC may account for their clinical features.

## Materials and methods

### Participants

The Affiliated Brain Hospital of Nanjing Medical University’s Medical Ethics Committee approved the current experiment, and each participant gave their informed consent in writing. Between October 2018 and August 2020, 25 drug-naive PD patients with TD (PD-TD) and 40 drug-naive PD patients with PIGD (PD-PIGD), and 37 healthy controls (HCs) were enlisted from the Department of Neurology of the Affiliated Brain Hospital of Nanjing Medical University. An experienced movement disorder specialist made a diagnosis for each PD subject according to the UK Parkinson’s Disease Society Brain Bank criteria (Hughes et al., 1992). The requirements for all subjects were as follows: (1) right-handed; (2) aged from 40 to 80; and (3) sighted or with corrected sighted and binaural hearing, meeting the assessment requirements and completing the examination. The inclusion criteria for PD patients were as follows: (1) the diagnostic criteria for PD met the Kingdom Parkinson Disease Society Brain Bank Criteria for idiopathic PD; (2) *de novo* PD patients without medication; and (3) Mini-Mental State Examination (MMSE) score ≥ 24. The following were the exclusion criteria for all subjects: (1) history of impaired consciousness; (2) history of manic episodes, schizophrenia, or other psychiatric diseases; (3) history of addiction to alcohol or drugs; (4) complications of severe brain, heart, kidney, liver, and hemopoietic system diseases; (5) contraindications to MRI scanning such as implantation of electronic and metallic devices; and (6) T2-weighted MRI showing vascular damage or cerebral infarction.

Clinical and imaging data were obtained prior to the initiation of any treatment. Clinical symptoms were assessed using rating scales before the MRI scan. The depression and anxiety severity was quantified using the 24-item Hamilton Depression Rating Scale (HAMD) and the 14-item Hamilton Anxiety Scale (HAMA). Cognitive function was evaluated with the Montreal Cognitive Assessment (MoCA) and Mini-Mental State Examination (MMSE). The PD severity was evaluated by using the unified PD rating scale (UPDRS) and the Hoehn and Yahr stage (H-Y). The classification of TD and PIGD subtypes was based on the approach adopted by Jankovic et al in 1990 (Jankovic et al., 1990). Using UPDRS II and III, the ratio of the mean UPDRS tremor scores (UPDRS-II item 16 and UPDRS-III items 20–21 divided by 8) to the mean UPDRS PIGD scores (UPDRS-II items 13–15 and UPDRS III items 29–30 divided by 5) was used to determine TD (ratio ≥1.5 or PIGD score = 0 and TD score >0), indeterminate (1.0 < ratios < 1.5 or both TD and PIGD scores = 0) and PIGD (ratio ≤1 or TD score = 0 and PIGD score >0) PD patients.

According to the above criteria, 10 intermediate patients, two PD-TD patients with poor MR image quality, two PD-PIGD patients, and three HCs with large head movement were excluded, 25 PD-TD patients (14M/11F), 40 PD-PIGD patients (20M/20F), and 37 age-, sex- and education-matched HCs (18M/19F) were finally included.

### Magnetic resonance imaging data acquisition and data preprocessing

MRI was performed using a 3T MRI scanner (Siemens, Verio, Germany). All participants were placed in the supine position with their heads immobilized by foam pads with a normal birdcage head coil to reduce head movement. The subjects were requested to remain as motionless as possible and to keep their eyes closed while staying awake and not thinking about anything. Axial anatomical images were acquired using a T1 fluid-attenuated inversion recovery sequence with parameters as follows: repetition time (TR) = 2,530 ms; echo time (TE) = 3.34 ms; field of view (FOV) = 256 × 256 mm; matrix = 256 × 192; slice thickness/gap = 1.33/0.5 mm; flip angle (FA) = 7 degrees; bandwidth = 180 Hz/px; 128 slices covered the whole brain, for image registration and functional localization. Functional images were then acquired in the same slice orientation using gradient-recalled echo-planar imaging pulse sequence. A total of 240 volumes were obtained (TR = 2,000 ms; TE = 30 ms; FOV = 220 × 220 mm; matrix = 64 × 64; thickness/gap = 3.5/0.63 mm; FA = 90 degrees; bandwidth = 2,232 Hz/px; slice numbers = 31).

The fMRI data were pre-processed using the Data Processing and Analysis for (Resting-State) Brain Imaging toolbox (DPABI 5.1^1^) using the MATLAB 2013b platform. The first 10 volumes of functional images were discarded to ensure signal equalization and participant adaptation to the scanning condition. The remaining images were corrected by realignment to account for head motion [five subjects (two PD-PIGD patients and three HC) were excluded due to excessive head motion (>3 mm or 3°)], normalized to Montreal Neurological Institute (MIN) standard space with diffeomorphic anatomical registration *via* exponentiated Lie algebra, resampled to a 3 × 3 × 3 mm voxel size, and were spatially smoothed with a 6-mm full width at half-maximum Gaussian kernel (Yan et al., 2013). In addition, the mean values of volume-based frame-wise displacements in TD-PD, PIGD-PD, and HC do not differ significantly (*p* <0.05).

### Independent component analysis and selection of networks of interest

The ICs were analyzed using Group ICA (the GIFT software^2^; projects/gift, version 3.0b). The procedures performed by this toolbox included three steps: (1) data reduction; (2) application of the ICA algorithm; and (3) back-reconstruction for individual-level components. In this study, 34 ICs were auto-estimated through the minimum description length (MDL) criteria, and Group ICA was carried out 20 times. Eleven meaningful components were identified as anatomically and functionally classical RSNs by visual inspection (Yeo et al., 2011; Shirer et al., 2012). The 11 ICs that correspond to the two sensorimotor networks (SMN), left executive control network (LECN), right executive control network (RECN), anterior SN (aSN), posterior SN (pSN), DAN, ventral attention network (VAN), DMN, VN, AN were then extracted from all participants. For each subject, the z-map of each component and its corresponding time courses (TC) represented the measures of intra-FC. Linear detrending, despiking, and temporal filtering were conducted for all TCs of the networks of interest before evaluating the inter-FC (Luo et al., 2014). We computed Pearson’s correlation coefficients of the TCs for each network pair among the SMN, LECN, RECN, aSN, pSN, DAN, VAN, DMN, VN, and AN and then used Fisher’s z-transformation to transform the coefficients into *z*-scores (Jafri et al., 2008). These transformed z-scores indexed the inter-FC of each network pair.

### Statistical analysis

Demographic data analysis was carried out using SPSS v25 software (IBM, United States). The comparison of the PD-TD and PD-PIGD groups was performed using a two-sample t-test or a Mann-Whitney U test. To compare the PD-TD, PD-PIGD, and HCs groups, one-way analyses of variance (ANOVA) or the Kruskal-Wallis H-test were used. In order to compare categorical variables such as gender, a chi-squared test was conducted.

To statistically evaluate the FC within each extracted ICN, it was computed voxel-wise from participants’ reconstructed spatial maps by one-sample *t*-test in SPM12 [*p* < 0.001, false discovery rate (FDR) corrected]. Connectivity comparisons within each ICN among PD-TD, PD-PIGD, and HCs groups were assessed using a design model of one-way ANOVA in SPM12, followed by a *post-hoc* two-sample *t*-test (*p* < 0.05, FDR corrected). The between-group differences of connectivity among selected ICNs were performed using ANOVA, and *post-hoc* two-sample *t*-test was applied to detect connectivity alterations between each pair of the three groups (*p* < 0.05, FDR corrected).

Spearman correlation analyses were performed to estimate the correlations between the identified connectivity abnormalities and the clinical symptom scores [including UPDRS-II, UPDRS-III, TD, PIGD, MMSE, MoCA, HAMD, HAMA, illness duration, and UPDRS III was divided into subscores for tremor (UPDRS-III items 20 and 21), rigidity (UPDRS-III item 22), bradykinesia (UPDRS-III items 23–26 and 31), axial symptoms (UPDRS-III items 27–30; Bohnen et al., 2011)] for the PD-TD and PD-PIGD patients in SPSS v25 software (*p* < 0.05, Bonferroni corrected).

SPSS v25 software was used for binary logistic regression analysis to test the diagnostic value of RSNs functional connectivity indicators in patients with TD and PIGD. RSNs functional indicators in univariate analysis were incorporated into the multi-factor model and were eliminated backward according to the likelihood ratio. The variable selection standard was *p* < 0.05. We assessed the receiver operating characteristic curve (ROC) and the area under the curve (AUC). To evaluate the predictive power, including accuracy, sensitivity, and specificity, of univariate and multivariate analysis models.

## Results

### Demographic and clinical characteristics

As shown in Table 1, there were no significant differences in disease duration, H-Y stage, UPDRS-II, UPDRS-III, rigidity, bradykinesia, and axial symptoms (*p* > 0.05) between the PD-TD and PD-PIGD groups, while no significant differences in age, gender, and education level (*p* > 0.05) among the three groups. In contrast, significant differences in the MMSE, MoCA, HAMD, and HAMA scores were observed among the three groups (*p* < 0.001); significant differences in the tremor, total TD, and total PIGD scores were observed between the PD-TD and PD-PIGD groups (*p* < 0.001).

**Table 1:** Demographic and clinical characteristics of the participants (mean ± SD).

**Variable**	**TD (*n* = 25)**	**PIGD (*n* = 40)**	**HC (*n* = 37)**	***p*-value**
Age (years)	61.84 ± 6.58	58.85 ± 7.41	59.30 ± 5.07	0.171^a^
Gender (M/F)	14/11	20/20	18/19	0.784^c^
Education (years)	9.04 ± 4.83	10.20 ± 3.30	11.07 ± 3.16	0.177^b^
Disease duration (months)	25.24 ± 15.85	20.54 ± 15.78	NA	0.362^e^
H-Y stage	1.74 ± 0.46	1.53 ± 0.53	NA	0.069^e^
UPDRS-II	8.04 ± 4.08	8.40 ± 3.73	NA	0.716^d^
UPDRS-III	25.48 ± 12.25	23.05 ± 11.32	NA	0.418^d^
Tremor	5.84 ± 4.11	1.55 ± 1.50	NA	<0.001^e^
Rigidity	5.68 ± 4.45	5.40 ± 3.64	NA	0.978^e^
Bradykinesia	10.64 ± 6.17	11.70 ± 6.97	NA	0.536^d^
Axial symptoms	2.16 ± 1.49	3.00 ± 1.74	NA	0.089^e^
TD	7.76 ± 4.52	2.48 ± 1.83	NA	<0.001^e^
PIGD	1.80 ± 1.29	3.08 ± 1.14	NA	<0.001^e^
MMSE	27.32 ± 1.97^g**^	27.15 ± 1.83^h***^	28.86 ± 1.29	<0.001^b^
MoCA	21.24 ± 3.75^g**^	22.35 ± 4.69^h**^	25.03 ± 3.23	<0.001^b^
HAMD	7.48 ± 4.51^g*^	12.13 ± 8.60^h***^	4.19 ± 4.90	<0.001^b^
HAMA	4.36 ± 3.62^f*^	8.05 ± 5.25^h***^	3.24 ± 4.13	<0.001^b^

UPDRS, Unified Parkinson’s Disease Rating Scale; H-Y stage, Hoehn and Yahr stage; TD, Tremor Dominant; PIGD, Postural Instability/Gait difficulty; MMSE, Mini-Mental State Examination; MoCA, Montreal Cognitive Assessment; HAMD, Hamilton Depression Rating Scale; HAMA, Hamilton Anxiety Rating Scale. a: one-way analysis of variance (ANOVA) tests; b: Kruskal-Wallis *H*-test; c: chi-square test; d: two-sample *t*-test; e: Mann-Whitney *U*-test; f: TD vs. PIGD; g: TD vs. HC; h: PIGD vs. HC. *p<0.05; **p<0.01, ***p<0.001. UPDRS-III was divided into subscores for tremor (UPDRS-III items 20 and 21), rigidity (UPDRS-III item 22), bradykinesia (UPDRS-III items 23–26 and 31), and axial symptoms (UPDRS-III items 27–30). TD scores (UPDRS-II item 16 and UPDRS III item 20, 21); PIGD scores(UPDRS-II item 13–15 and UPDRS-III item 29, 30).

### Identification of network of interests

**Figure 1:**
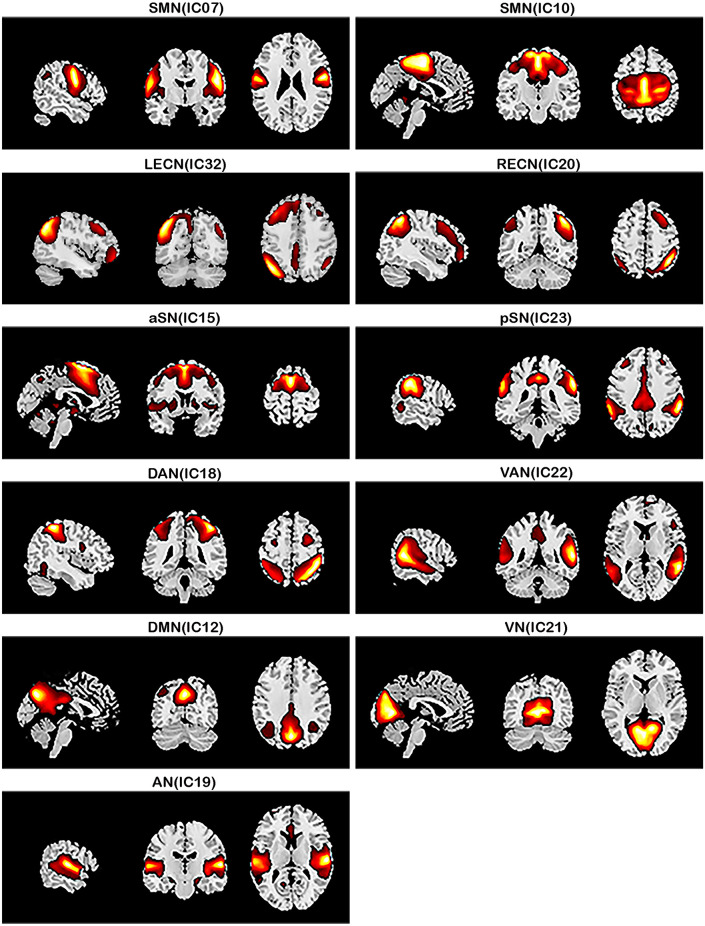
Large-scale networks including the 11 resting-state networks (RSNs) identified by independent component analysis (one-sample *t*-test, *p* < 0.001, FDR corrected). Sensorimotor network, SMN (IC07/IC10); left executive control network, LECN (IC32); right executive control network, RECN (IC20); anterior salience network, aSN (IC15); posterior salience network, pSN (IC23); dorsal attention network, DAN (IC18); ventral attention network, VAN (IC22); default mode network, DMN (IC12); visual network, VN (IC21); auditory network, AN (IC19).

The respective spatial patterns of 11 networks of interests for the three groups (PD-TD, PD-PIGD, and HCs) were revealed by the one-sample *t*-test (Figure 1; *p* < 0.001, FDR corrected), including the SMN (IC07/10), LECN (IC32), RECN (IC20), aSN (IC15), pSN (IC23), DAN (IC18), VAN (IC22), DMN (IC12), VN (IC21), and AN (IC19).

### Intra-network connectivity analysis

No significant differences in intra-network FC were observed among the three groups.

### Inter-network connectivity analysis

**Figure 2:**
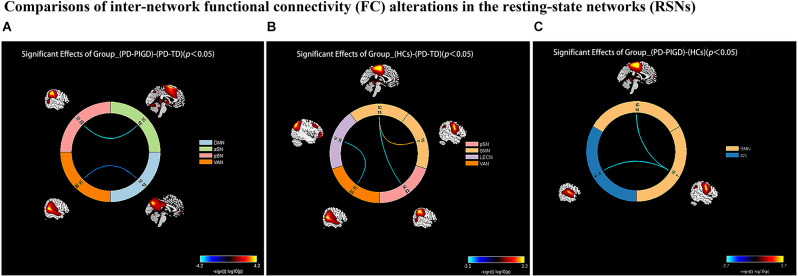
Comparisons of inter-network functional connectivity (FC) alterations in the resting-state networks (RSNs) between the Parkinson’s disease (PD) with tremor dominant (TD) and postural instability and gait difficulty (PIGD) groups. **(A)** Compared with the PD-TD group, the PD-PIGD group exhibited a decreased connectivity between anterior salience network (aSN) and posterior salience network (pSN), and between ventral attention network (VAN) and default mode network (DMN; *p* < 0.05, FDR corrected). **(B)** Compared with the HCs group, the PD-TD group exhibited a decreased connectivity between two sensorimotor (SMN) subnetworks (IC7 and IC10); but an increased connectivity between left executive control network (LECN) and VAN, and between the SMN subnetwork (IC10) and pSN (*p* < 0.05, FDR corrected). **(C)** Compared with the HCs group, the PD-PIGD group exhibited a decreased connectivity between two SMN subnetworks (IC7 and IC10), and between the SMN subnetwork (IC7) and auditory network (AN; *p* < 0.05, FDR corrected).

Compared with the PD-TD group, the PD-PIGD group showed decreased FC between aSN and pSN, and between VAN and DMN (*p* < 0.05, FDR corrected; Figure 2A). Compared with HCs, both PD-TD and PD-PIGD patients displayed decreased FC between two SMN subnetworks (IC7 and IC10), while PD-TD patients exhibited increased FC between the SMN subnetwork (IC10) and pSN, and between LECN and VAN (*p* < 0.05, FDR corrected; Figures 2B,C). Furthermore, PD-PIGD patients demonstrated reduced FC between the SMN subnetwork (IC7) and AN (*p* < 0.05, FDR corrected; Figure 2C).

### Correlation analysis of functional connectivity with neuropsychological test scores and clinical characteristics

**Figure 3:**
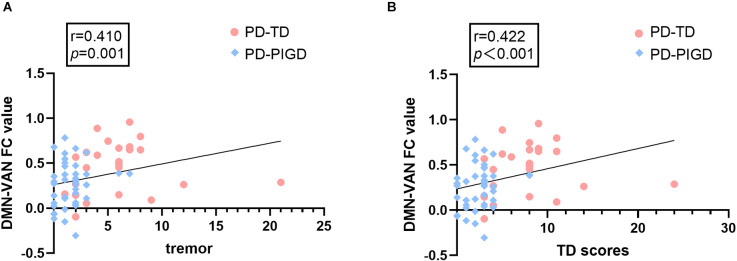
The correlations between internetwork connectivity and tremor and TD scores in PD patients. **(A)** FC between the default mode network (DMN) and ventral attention network (VAN) shows a significant positive correlation with tremor scores (*p* = 0.001). **(B)** FC between DMN and VAN shows a significant positive correlation with TD scores (*p* < 0.001).

The Spearman correlation analysis demonstrated that in PD-TD and PD-PIGD groups, the FC alterations between the VAN and DMN were positively correlated with tremor scores (*r* = 0.410, *p* = 0.001) and TD scores (*r* = 0.422, *p*<0.001; Figure 3).

### Classification of TD and PIGD subtypes using the ROC analysis

**Figure 4:**
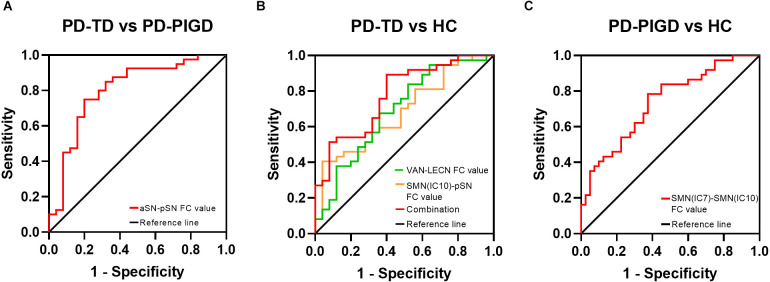
Receiver operating characteristic (ROC) curves for predicting PD-TD and PD-PIGD patients by neuroimaging biomarkers of internetwork connectivity. **(A)** ROC curve showing the classification of TD and PIGD. **(B)** ROC curve showing the classification of TD and HCs. **(C)** ROC curve showing the classification of PIGD and HCs.

ROC analysis was performed to compute the value of changed indicators to categorize PD-TD and PD-PIGD. In the PD-TD and PD-PIGD groups, the AUC value of FC between aSN and pSN was 0.803 with *p* < 0.001, with sensitivity = 80%, and specificity = 75%. In the PD-TD and HCs groups, The AUC value of FC between VAN and LECN was 0.690 with *p* = 0.012, with the sensitivity = 48.0%, specificity = 83.8%, the AUC value of FC between SMN subnetwork (IC10) and pSN was 0.683 with *p* = 0.015, with the sensitivity = 96%, specificity = 40.5%, and the optimal classification method was based on the integration of multiple indexes, with an AUC value of 0.777, *p* < 0.001, sensitivity = 89.2%, and specificity = 60% for the combined multiple indicators. In the PD-PIGD and HCs groups, the AUC value of FC between two SMN subnetworks was 0.736 with *p* < 0.001, with sensitivity = 78.4%, and specificity = 62.5% (Figure 4).

## Discussion

This study thoroughly investigated the FC alterations within and between broad-scale networks associated with PD motor subtypes. No significant differences in intra-network connectivity were detected among the three groups. Impaired inter-network FC was found to be the cause of TD and PIGD subtypes dysfunction, which was consistent with previous studies on PD network connection injury. Previous studies have found that poor brain network integration is a major driver of PD dysfunction (Gottlich et al., 2013; Baggio et al., 2014; Peraza et al., 2017; Gratton et al., 2019). Our findings provide the groundwork for understanding the neurophysiological mechanisms of TD and PIGD in PD, as well as establishing a differential diagnosis.

In this study, compared with the PD-TD group, the PD-PIGD group exhibited decreased FC between the aSN and pSN, and between the VAN and DMN. Notably, VAN-DMN FC value was positively correlated with TD and tremor scores, and the aSN-pSN value provided the basis for the best-fitting classification models for the TD and PIGD subtypes. The activation of the ventral attention network, which is associated with stimulus-driven attention orientation, occurs when unexpected stimuli are detected and attention is shifted (Vossel et al., 2014). The VAN specifically participates in top-down regulated attentional selecting and bottom-up attentional processing through dynamic interaction with DAN, therefore controlling externally oriented attention, emotional regulation, and cognition (Chica et al., 2013). Additionally, in terms of resting-state brain connectivity, VAN and DMN are anti-correlated (Fox et al., 2005). According to studies, PD-related fatigue is linked to alterations in the attention network (Zhang et al., 2017). Our results suggest that the decrease of antagonism between VAN and DMN in PD patients is related to tremor symptoms, also demonstrated that inadequate connectivity in PD patients is associated with behavioral disorders. Among the many complex functional networks of the human brain, the triple-network model is considered as the core network of human cognition and emotion, consisting of DMN, ECN, and SN. The DMN and ECN are two modes of passive and active information processing in the human brain. Switching between the two modes is realized by the SN. The SN plays important roles in maintaining the dynamic balance between the ECN and DMN, integrating sensory and motor information, and processing bottom-up information (Seeley et al., 2007; Menon, 2011; Huang et al., 2020). In general, the aSN is more related to cognitive and emotional functions, while the pSN contributes more to autonomic and sensory functions. Previous studies have found that the interruption of the connection between significant network and executive control network may lead to the occurrence of PD depression (Huang et al., 2020). We found that the TD and PIGD subtypes had distinct FC between the aSN and pSN, which led to defective dynamic regulation of the triple-network model made up of the DMN, ECN, and SN. This finding could explain why their non-motor symptoms progressed differently.

In this study, the FC between the two SMN subnetworks (IC7 and IC10) was lower in PD-TD and PD-PIGD patients compared to HCs. The SMN is essential for planning and carrying out motor actions (Esposito et al., 2013), including the sensorimotor cortex, secondary sensorimotor cortex, and supplementary motor area to promote voluntary movement (Smith et al., 2009). Systematic evaluations of SMN are insufficient, and evidence for network alterations of SMN in PD patients is inconsistent. Our findings indicate disruption of motor system injury in *de novo* PD patients with TD and PIGD subtypes, which is consistent with previous studies. The medical OFF status of PD patients was found to have decreased FC between the two subnetworks of the SMN, and dopaminergic treatment can partially compensate for SMN decoupling (Caspers et al., 2021). The motor subtype system showed instability, with 18% of patients with TD and 39% of patients with PIGD becoming different subtypes after 1 year (Simuni et al., 2016). The motor subtype system demonstrated instability, with distinct subtypes emerging in 18% of TD patients and 39% of PIGD patients after 1 year.

Multiple studies have manifested that PIGD patients exhibit a quicker cognitive decline, higher risk of depression, poorer quality of life, and faster disease progression than TD patients (Burn et al., 2012; Ba et al., 2016; Wen et al., 2018). Because of the negative consequences seen in PIGD, TD is regarded as a benign subtype (Helmich et al., 2012). Early in the course of the disease, TD patients displayed regionally increased fractional anisotropy but decreased diffusivities, suggesting neural reorganization to counteract PD pathology, while PIGD patients displayed more white matter degradation, despite having similar disease stages and duration (Wen et al., 2018). Based on previous work, our study is the first to demonstrate potential neural compensation in the TD subtype by FC enhancement between the SMN (IC10) and aSN, and between LECN and VAN. The ECN is the only lateralized brain network, which is divided into LECN and RECN, and primarily engaged in top-down attention, cognitive activities, emotion and behavioral control (Kaiser et al., 2015). The LECN is unique to humans and has phonetic semantic and memory functions, while the functions of RECN include sensory-soma-pain, working memory, and inhibition (Seeley et al., 2007; Lerman et al., 2014). Previous research has shown that PD patients increase their attentional resources to make up for the decline in motor automaticity. This attention strategy has the effect of reducing the amount of time that movement is mediated by automatic processes and increasing the amount of time that it is mediated by attentional motor control processes (Lu et al., 2010). Therefore, we suggest the compensatory mechanism for TD patients is achieved by enhancing FC between the LECN and VAN to increase attention resources.

PD patients with PIGD subtype are characterized by relatively extensive neural activity abnormalities, particularly in motion-related areas (Jiang et al., 2016). Dopaminergic drugs are used to lessen motor symptoms, while adjuvant therapies, such as acoustic-based non-pharmacological interventions, are utilized as a supplement to conventional drug treatments. Numerous studies have shown that rhythmic auditory stimulation (RAS) can help Parkinson’s disease sufferers walk more naturally (Lee et al., 2012; Benoit et al., 2014; Leuk et al., 2020). For instance, walking to an audio metronome can reduce timing variability, increase walking speed, and lengthen strides (Benoit et al., 2014). Because of the intimate neuronal connections between the auditory and motor regions, RAS is thought to have a stabilizing impact (Hove and Keller, 2015). In this study, the reduced FC between SMN subnetwork (IC7) and AN was observed in the PD-PIGD subtype at resting state, which provides a neuropathological basis for the interaction between acoustic therapy and improvement of motor symptoms in PD-PIGD patients, and also indicated that early acoustic therapy is more necessary for PD patients with PIGD subtype.

Nevertheless, this study contains several limitations. First, due to the strict inclusion criteria for *de novo* PD patients without drug interference, the sample size of this study is relatively small. A larger sample size will be needed to confirm the findings in the future. Second, de novo PD patients typically experience mild motor abnormalities and non-motor symptoms, and a certain proportion of motor subtype changes occur in them as the disease progression (Simuni et al., 2016; Lee et al., 2019). Longitudinal follow-up assessment should be used in future studies to evaluate motor subtypes at varied clinical stages. Third, while only applicating neural network connection as a biomarker is limited, it is anticipated that our patient cohort will also be affected by genetic factors, multimodality imaging, environmental factors, or plasma measures.

## Conclusions

In summary, the current study demonstrated that the altered FC between aSN and pSN can be an imaging marker to distinguish TD from PIGD. We for the first time disclosed that the TD subtype compensated by increasing attention resources and the PIGD subtype showed reduced FC between SMN and AN. Our findings provide a basis for differential diagnosis and precision treatment of PD motor subtypes.

## Data availability statement

The original contributions presented in the study are included in the article, further inquiries can be directed to the corresponding author.

## Ethics statement

The study was reviewed and approved by the Medical Ethics Committee of the Affiliated Brain Hospital of Nanjing Medical University. The participants provided their written informed consent to participate in this study.

## Author contributions

WL conceived and organized the research. LY, JX, YW, and GZ collected the data. QW analyzed the data and wrote the manuscript. MY and JX made manuscript revision. WL authorized the final version of the manuscript for publication. All authors contributed to the article and approved the submitted version.

## Footnotes

^1^ http://rfmri.org/dpabi

^2^ https://www.nitrc.org/
